# Revisiting the Dynamic Response of Chinese Price Level to Crude Oil Price Shocks Based on a Network Analysis Method

**DOI:** 10.3390/e24070944

**Published:** 2022-07-07

**Authors:** Qingru Sun, Ze Wang, Nanfei Jia

**Affiliations:** 1 School of Economics, Hebei University, Baoding 071002, China; sunqingru@hbu.edu.cn; 2 International Academic Center of Complex Systems, Beijing Normal University, Zhuhai 519087, China; 3 School of Systems Science, Beijing Normal University, Beijing 100875, China; 4 School of E-Business and Logistics, Beijing Technology and Business University, Beijing 100048, China; nanfei.jia@btbu.edu.cn

**Keywords:** crude oil price shocks, price indices, indirect influence, network analysis method, impulse response function

## Abstract

Crude oil price shocks have led to a fluctuation in commodity prices through the industrial chain and supply–demand relationships, which can substantially influence a country’s economy. In this paper, we propose a transmission model of oil price shocks to Chinese price levels and explore the direct and indirect impacts of crude oil price shocks on various Chinese price indices, combining the Granger causality test, impulse response function, and network analysis method. The empirical data are the Brent, WTI, Dubai, and Daqing spot crude oil prices and eight categories of Chinese price indices from January 2011 to March 2020. We found the following results: (1) Consumer price index (CPI) and the price index for means of agricultural production (MAPPI) cannot be directly impacted by crude oil price fluctuations, while they could be indirectly affected. (2) The duration and degree of the impacts of oil prices on each price index vary, and the export price index (EPI) is the most significantly affected. (3) The proportion of the indirect impact in the total impact of crude oil price shocks ranges from 0.03% to 100.00%. Thus, indirect influence cannot be ignored when analyzing the influence of crude oil price fluctuation on Chinese price level.

## 1. Introduction

Crude oil is a primary raw resource in the economy [1]. China’s dependence on foreign crude oil shows an increasing trend (reaching 72% in 2021), making the Chinese economy sensitive to international crude oil price fluctuations that is prominently reflected in the Chinese price level [2,3,4]. The fluctuations in crude oil prices could directly affect the price of products. For example, the prices of import and export goods are easily affected due to the impact of crude oil price fluctuations on international trade costs. The prices of means of production are also easily affected because crude oil acts as the primary raw material for producing. Besides, the directly affected goods could further transmit the price fluctuations to other goods through supply–demand relationships and substitution relationships among commodities [5]. For example, due to the long and complex crude oil industrial chain, the price fluctuations of means of production could continue to influence more and more goods, such as agricultural products, industrial products, and consumer goods. In this paper, we define that there is an indirect impact from crude oil price fluctuations on such agricultural products, industrial products, and consumer goods. Therefore, it is vital to research the direct and indirect influences of crude oil price fluctuation on various Chinese commodities, providing a new perspective for China to improve its ability to prevent risk from cost-push inflation.

The existing methods used to study the transmission mechanism of the international oil price on price level are the calculable general equilibrium models [6,7], input–output price model [8], and econometric models based on time series [9,10,11,12,13]. However, first, the general equilibrium models have high data and model settings requirements. Second, although the input–output price model is widely used to analyze the direct and indirect impacts of energy price changes on the prices of all sectors’ products [8,14], the input–output table of China is released every five years; thus, we cannot obtain the newest data to analyze the influence of oil price fluctuation on price levels promptly. Third, time-series data have the advantages of high frequency and good timeliness. Hence, the econometric modeling methods based on time series, such as structural vector autoregression, Granger causality test, and impulse response function, are relatively flexible. However, these econometric methods have limitations for analyzing the indirect effects of one variable on other variables. Therefore, a new analysis framework is inevitable to develop.

This paper proposes a novel framework to analyze the direct and indirect influences of crude oil price shocks on Chinese price levels, combining the network analysis method and econometric methods, namely, the Granger causality test and impulse response function. We select the Brent, WTI (West Texas Intermediate), and Dubai spot crude oil prices to represent international crude oil prices and the Daqing spot crude oil price to represent Chinese crude oil prices. Eight Chinese price indices are selected to represent different commodity price levels. First, the Granger causality test is used to reveal the fluctuation transmission relationships among crude oil price and price indices. Then, we analyze the direct effects of crude oil price shocks on every price index based on the impulse response function and extract the response values with their corresponding time to explore the evolution of the direct impact degree and the duration of the impact. Third, based on the results of the Granger causality test and impulse response function, this paper proposes a transmission model of oil price shocks to Chinese price levels using the network analysis method and analyzes the indirect influence on price indices over time. Finally, we calculate the total influence of crude oil price shocks on price indices and compare the indirect impacts with total impacts. Our research provides closer observations that consider the direct and indirect effects of crude oil price shocks on each category of price index. This could produce a more comprehensive picture of the transmission mechanism of crude oil price fluctuations on Chinese price levels and could provide policy implications for China to improve its ability to prevent risk from imported inflation.

The paper is organized as follows. Section 2 introduces the data and methods. Section 3 presents the empirical results. Conclusions are presented in Section 4.

## 2. Literature Review

The impact of crude oil price fluctuation on inflation and GDP has always been an important issue [15,16,17,18]. The price indices are widely used to represent inflation. According to the research aims, the related existing studies can be divided into the following three categories.

First, the heterogeneous impacts of crude oil price fluctuations on inflation in different economies have been proved [19,20,21]. Researchers revealed that oil market volatility strongly and negatively affects real economic growth. Specifically, it exerts more pressure on inflation in Saudi Arabia and the United Kingdom [22,23] but less influence on the ASEAN-5 economies (Thailand, Malaysia, Singapore, the Philippines, and Indonesia) in the long run [24]. Oil price could exert a more significant impact on inflation of net oil-importing countries than their oil-exporting counterparts in the long run [25]. Choi, S. et al. studied the effects of international oil price fluctuations on the inflation of 72 advanced and developing economies and found that a 10% increase in crude oil price could cause domestic inflation by increasing approximately 0.4% on average, with the influence vanishing after two years [26]. They explained that the share of transport in the CPI basket and energy subsidies are the most robust factors in explaining cross-country variations in the effects of oil price shocks. As for China, a net importer of crude oil, crude oil price fluctuations are more likely to influence its inflation significantly [18,19]. The pass-through effects of global oil price on China’s producer prices index (PPI) and consumer prices index (CPI) have been demonstrated [2], and demand shocks that are specific to the crude oil market contribute the most to the fluctuations in China’s inflation [27,28].

Second, the non-linear effects of oil price on inflation have been revealed based on nonlinear ARDL methods [9,29], the Markov Regime Switching Vector Autoregressive model [30], and non-linear causality tests [31]. Lacheheb and Sirag [32] proved the nonlinear impacts of oil price on inflation in Algeria. Specifically, there is a significant relationship between the rise in oil price and inflation rate. However, there is no significant relationship between the decline in oil prices and inflation. Long and Liang [2] studied the pass-through effects of international crude oil price on China’s CPI and PPI and found that the long-term impacts of the oil price reduction on China’s inflation are lower than the oil price increases on inflation. However, researchers found that the impacts on African OPEC member countries’ inflation were found to be more significant when the oil prices decreased [33].

Third, the impacts of oil price fluctuations on various price indices are also different [5,34,35]. For example, in Malaysia, oil price changes could directly cause higher import and production prices through cost channels but have a limited direct effect on consumer prices [29]. Researchers also revealed that an increase in oil price had a stronger effect on the PPI than the CPI in Indonesia and Thailand [36]. Similar effects of oil price shocks on CPI and PPI in China were also proven, i.e., the demand shocks of oil price had significant effects on China’s PPI, while the impacts failed to be transmitted to China’s CPI [28].

The above literature mainly focuses on the transmission mechanism from oil price fluctuation to CPI or PPI using econometric models from a macro perspective. However, other macro price indices could also represent the price level of an economy to a certain degree, such as the retail price index (RPI), the purchasing price index of raw material, fuel, and power (PPIRM), and so on. More importantly, there are complex links between these price indices. Previous studies ignored the transmission relationships between crude oil and other price indices, such as RPI and PPIRM. Besides, because of the complicated transmission relationships among price indices, crude oil price fluctuations could also indirectly impact Chinese commodities. Existing studies paid little attention to the indirect impact of oil price fluctuation on the price level. What are the direct and indirect effects of oil price fluctuations on price indices? How do these effects change over time? How long do the effects last? By answering these questions, we can deepen the understanding of the transmission mechanism of oil prices to the Chinese price level.

## 3. Data and Methods

### 3.1. Data

We select the crude oil spot price of WTI, Brent, Dubai, and Daqing (China) as empirical objects. The data are monthly month-on-month data obtained from the Wind Database (http://www.wind.com (accessed on 1 March 2022)) and the Energy Information Administration (http://www.eia.gov (accessed on 1 March 2022)). Since international crude oil trading price is based on the price of Brent, WTI, and Dubai crude oil, the monthly spot prices of Brent, WTI, and Dubai crude oil are selected to represent the international crude oil price level. The monthly spot price of Daqing crude oil is chosen to represent the Chinese crude oil price level. The study period is from January 2011 to March 2020; thus, there are 110 observations for each time series during the sample period. Then, we calculate the log return of the crude oil prices. The price indices we use in this paper are CPI, PPI, RPI, PPIRM, price index for means of agricultural production (MAPPI), corporate goods price indices (CGPI), the export price index (EPI), and the import price index (MPI), which reflect the Chinese price levels. The data period is from January 2011 to March 2020, downloaded from the Wind Database. We process the time series with a first-order difference. Based on the ADF test, all the variables are stationary.

### 3.2. The Measure of the Direct and Indirect Effects

#### 3.2.1. The Measure of Direct Effects

The Granger causality test is a common method for determining whether there is causality between two variables. The impulse response function can analyze the dynamic effects (namely the influence degree) of variable changes on the overall system, i.e., how the volatility of one variable is transmitted to other variables over time, thus leading to fluctuations in other variables. We take the Brent crude oil as an example; the measure of the direct effect on each price index includes the following five main steps.

Step 1: Building the VAR model

If the time series is stationary, we construct a bivariate VAR model that pairs the crude oil price and each price index. In a VAR model, all variables are treated as endogenous variables, and it can estimate the dynamical interaction between variables. The mathematical expression of the VAR model is as follows:

(1) $$y_{t}=\Phi_{1}y_{t-1}+\Phi_{2}y_{t-2}+\cdot \cdot \cdot +\Phi_{p}y_{t-p}+e_{t},\;t=1,\;2,\cdot \cdot \cdot ,\;T,$$

where $y_{t}$ is a k-dimension column vector of an endogenous variable. Φ is a coefficient matrix of k × k. *p* is the lag order, and *T* is the simple length. $e_{t}$ is a *k*-dimension white noise vector. The Akaike information criterion is used to determine the value of *p*.

Step 2: Granger causality test

The Granger causality test is based on precedence and predictability. According to [37], there are nine variables; thus, we carry out $\frac{9*\left(9-1\right)}{2}=36$ Granger causality tests for each pair of variables. The results could be described as a matrix G, shown in Equation (2).

(2) $$G=\left[\begin{array}{cccccc} 0 & \cdots & \cdots & G_{1j} & \cdots & G_{1n}\\ \vdots & \ddots & \ddots & \vdots & \ddots & \vdots \\ G_{i1} & \cdots & \cdots & G_{ij} & \cdots & G_{in}\\ \vdots & \ddots & \ddots & \vdots & \ddots & \vdots \\ \vdots & \ddots & \ddots & \vdots & \ddots & \vdots \\ G_{n1} & \cdots & \cdots & G_{nj} & \cdots & 0 \end{array}\right]\;$$

where $G_{ij}$ represents the causal relationship (or transmission relationships) from variable *i* to variable *j*, and *I* ≠ *j*. *n* is the number of variables and equals 9 in this paper. We set the first variable with the crude oil price, i.e., “1” represents crude oil, and “2” to “9” represent price indices. Choosing a suitable significant level is crucial to the result of the Granger causality test, and this paper chooses the significance level of 0.05 according to the existing studies [38,39,40]. If there is a significant causal relationship from *i* to *j*, then $G_{ij}\;$= 1; otherwise, $G_{ij\;}$= 0.

Step 3: Judging whether the VAR model is stationary

The bivariate VAR model, constructed in step 1, needs to be tested by the AR root. If the reciprocal of the absolute value of all roots in the VAR model is less than 1, the VAR model is stable in the unit circle. If the model is unstable, some results may not be valid (e.g., the standard error of the impulse response function).

Step 4: Building the impulse response function

If the VAR model is stable, we build an impulse response function, which is used to measure the impact of a positive shock of one standard deviation from an endogenous variable on all endogenous variables in the VAR model during the current and future periods. The impulse response function can be expressed as follows:

First, the VAR model constructed in step 1 can also be expressed as Equation (3).

(3) $$y_{t}=(I_{k}+A_{1}L+A_{2}L^{2}+\cdot \cdot \cdot )e_{t},\;t=1,\;2,\cdot \cdot \cdot ,\;T,$$

where *L* is a lag operator, and $I_{k}$ is an identity matrix with *k*-dimensions. The *i*th variable of $y_{t}$ is $y_{it}$, which is as follows:

(4) $$y_{it}=\sum_{j=1}^{k}\left(r_{ij}^{\left(0\right)}e_{jt}+r_{ij}^{\left(1\right)}e_{j\left(t-1\right)}+r_{ij}^{\left(2\right)}e_{j\left(t-2\right)}+\cdots \right),\;t=1,\;2,\cdots ,T.$$

Supposing that there is a standard deviation to $y_{1}$ at time 0,

(5) $$e_{1t}=\{\begin{array}{c} 1,\;t=0\;\;\;\;\;\;\;\;\;\;\;\;\;\;\\ 0,\;t=1,\;2,\;3,\cdots \end{array},\;e_{it}=0;\;\;t=0,1,2,\;\cdots \;and\;i\neq 1.$$

Therefore, the impulse response of $y_{j}$ to $y_{1}$ is $r_{1j}^{\left(0\right)},\;r_{1j}^{\left(1\right)},\;r_{1j}^{\left(2\right)},\;\cdots$, where $r_{1j}^{\left(1\right)}$ represents the response value of $y_{j}$ in the first month after the shock of $y_{1}$.

The equation of the response of $y_{j}$ on time *q* after the shock of $y_{i}$ can be expressed as follows:

(6) $$r_{ij}^{\left(q\right)}=\frac{\partial y_{i,t+q}}{\partial e_{jt}},\;q=0,\;1,\;2,\;\cdots ,\;N;\;t=1,\;2,\;\cdots ,\;T.$$

We implement the impulse response function between each pair of price indices and between crude oil and each price index and extract the impulse response values in each month, which can be shown in a matrix $R^{\left(q\right)}$.

(7) $$R^{\left(q\right)}=\left[\begin{array}{cccccc} r_{11}^{\left(q\right)} & \cdots & \cdots & r_{1j}^{\left(q\right)} & \cdots & r_{1n}^{\left(q\right)}\\ \vdots & \ddots & \ddots & \vdots & \ddots & \vdots \\ r_{i1}^{\left(q\right)} & \cdots & \cdots & r_{ij}^{\left(q\right)} & \cdots & r_{in}^{\left(q\right)}\\ \vdots & \ddots & \ddots & \vdots & \ddots & \vdots \\ \vdots & \ddots & \ddots & \vdots & \ddots & \vdots \\ r_{n1}^{\left(q\right)} & \cdots & \cdots & r_{nj}^{\left(q\right)} & \cdots & r_{nn}^{\left(q\right)} \end{array}\right]$$

Step 5: Measure the direct effects

Combining the results of Granger causality and impulse response function, we can obtain a new matrix $GR^{\left(q\right)}$ revealing the direct effects among variables at time *q* after a shock of a certain variable.

(8) $$GR^{\left(q\right)}=G⊙R^{\left(q\right)}=\left[\begin{array}{cccccc} G_{11}*r_{11}^{\left(q\right)} & \cdots & \cdots & G_{1j}*r_{1j}^{\left(q\right)} & \cdots & G_{1n}*r_{1n}^{\left(q\right)}\\ \vdots & \ddots & \ddots & \vdots & \ddots & \vdots \\ G_{i1}*r_{i1}^{\left(q\right)} & \cdots & \cdots & G_{ij}*r_{ij}^{\left(q\right)} & \cdots & G_{in}*r_{in}^{\left(q\right)}\\ \vdots & \ddots & \ddots & \vdots & \ddots & \vdots \\ \vdots & \ddots & \ddots & \vdots & \ddots & \vdots \\ G_{n1}*r_{n1}^{\left(q\right)} & \cdots & \cdots & G_{nj}*r_{nj}^{\left(q\right)} & \cdots & G_{nn}*r_{nn}^{\left(q\right)} \end{array}\right]$$

where ⊙ means the Hadamard product. Thus, if $G_{ij}*r_{ij}^{\left(q\right)}$ does not equal 0, it reveals the direct impact degree of variable *i* on variable *j* in the *q*th month when there is a Granger causality from *i* to *j*. Then, the direct effects of crude oil price shocks on price indices are $GR_{1s}^{\left(q\right)},\;s=2,3,4,\ldots ,\;9$. Therefore, combining the Granger causality test and the impulse response function, we could not only identify the significant transmission relations but also obtain the direct influence degree among variables.

**Figure 1:**
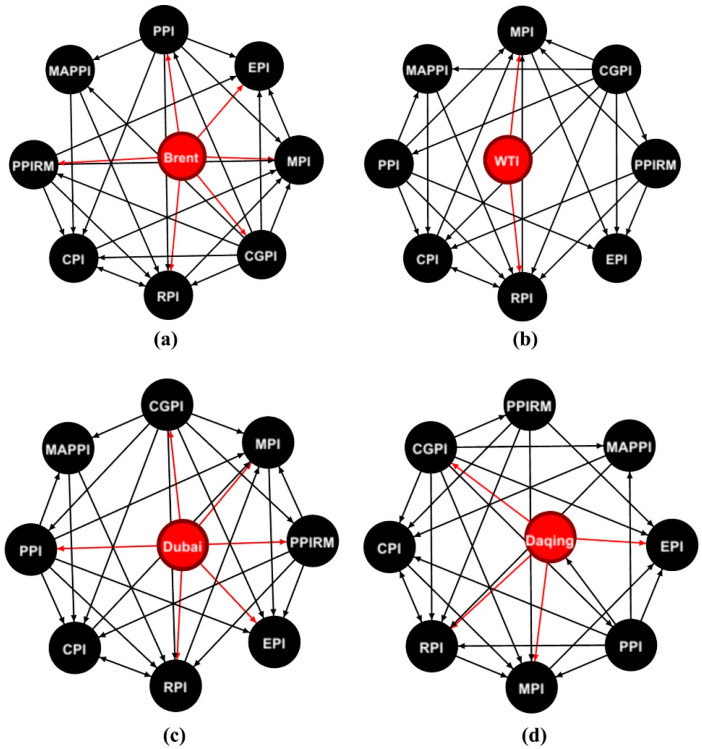
The transmission networks of four oil price shocks to Chinese price indices (the red nodes represent crude oil, and the back nodes represent price indices. (**a**–**d**) represents the transmission network of Brent, WTI, Dubai, and Daqing crude oil price shocks to Chinese price indices, respectively).

Then, we propose a transmission network of oil price shocks to Chinese price levels (TNOP) with variables as nodes and transmission relationships as edges. The response values are the weight of the edges. Therefore, the network can be described as Equation (9).

(9) $$TNOP=\left(V,E,W\right)=GR^{\left(q\right)}$$

where *V* represents the node set, and *E* and *W* represent the edge set and weight set, respectively. If $G_{ij}*r_{ij}^{\left(q\right)}$ does not equal 0, then there is an edge from variable *i* to *j*. Since the weights of the edges vary over time, we only display the unweighted transmission networks of four oil price shocks to various price indices, as shown in Figure 1. The directional red lines represent the transmission relationships from crude oil price shock to price indices, i.e., the direct impact from crude oil to price indices. It is observed that not all the price indices could be directly affected by crude oil. Furthermore, the transmission relationships among price indices are complicated.

#### 3.2.2. The Measure of Indirect Effects

**Figure 2:**
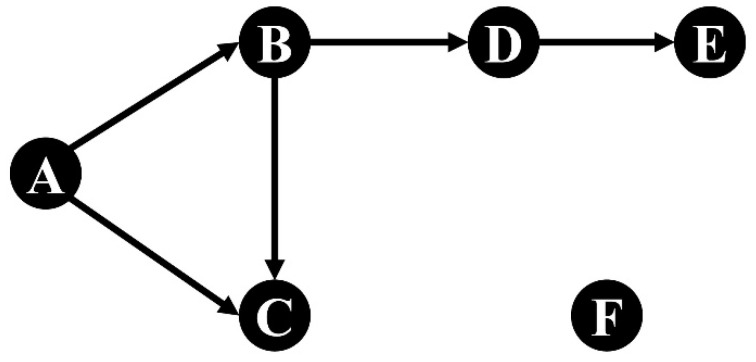
A simple unweighted transmission network.

In this paper, we explore the indirect effect of crude oil price shocks on Chinese price level based on the TNOP. Since the sample data are monthly data, this paper assumes that the transmission of price shocks between two variables needs one month. Figure 2 is a simple transmission network. There is an edge from A to B and from A to C; thus, the price fluctuations of A could be directly transmitted to B and C in the first month after A is shocked. Due to the transmission relationships from B to C and from B to D, the affected B could further impact C and D; thus, there is an indirect impact from A to C and from A to D in the second month after A is shocked. Similarly, the influence of the price fluctuations of A could indirectly impact E in the third month. Thus, the indirect effects arise from the second month after a onetime positive shock to A. Furthermore, there is no transmission path from A to F; thus, the price fluctuations of A could not affect F.

Since $GR^{\left(q\right)}$ is regarded as a direct impact matrix at time *t*, $GR^{\left(0\right)}$ and $GR^{\left(1\right)}$ are the direct impact matrices for the current month and the first month after the shock, respectively. $GR^{\left(0\right)}\times GR^{\left(1\right)}$ is the indirect impact matrix in the first month, and $GR^{\left(0\right)}\times GR^{\left(1\right)}\times GR^{\left(2\right)}$ is the indirect impact matrix in the second month. Generally, the indirect impact matrix is defined as Equation (10).

(10) $$IR^{\left(q\right)}=GR^{\left(0\right)}\times GR^{\left(1\right)}\times \cdots \times GR^{\left(q\right)}=\left[\begin{array}{cccccc} ir_{11}^{\left(q\right)} & \cdots & \cdots & ir_{1j}^{\left(q\right)} & \cdots & ir_{1n}^{\left(q\right)}\\ \vdots & \ddots & \ddots & \vdots & \ddots & \vdots \\ ir_{i1}^{\left(q\right)} & \cdots & \cdots & ir_{ij}^{\left(q\right)} & \cdots & ir_{in}^{\left(q\right)}\\ \vdots & \ddots & \ddots & \vdots & \ddots & \vdots \\ \vdots & \ddots & \ddots & \vdots & \ddots & \vdots \\ ir_{n1}^{\left(q\right)} & \cdots & \cdots & ir_{nj}^{\left(q\right)} & \cdots & ir_{nn}^{\left(q\right)} \end{array}\right],q=1,2,3,4,\cdots$$

where $IR^{\left(q\right)}$ represents the indirect impact matrix in the *q*th month; $ir_{ij}^{\left(q\right)}$ is the indirect impact of *i* on *j* in time *q*. Thus, the indirect influence of crude oil shocks on each price index in the *q*th month is $ir_{1j}^{\left(q\right)},\;j=2,\;3,\;\cdots ,\;9$.

## 4. Empirical Results and Discussion

### 4.1. The Direct Effects of Crude Oil Price Shocks

**Figure 3:**
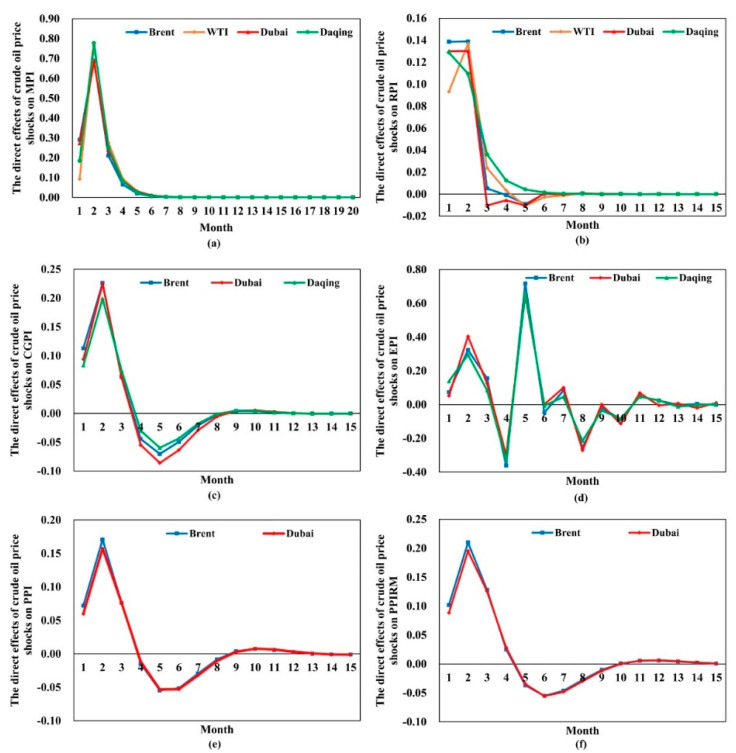
The direct impacts of crude oil price shocks on each price index. The *X*-axis represents the time from the first month to the fifteenth month. (**a**–**f**) represent the direct effects of crude oil price shocks on MPI, RPI, CGPI, EPI, PPI, and PPIRM, respectively. We draw another figure to compare the response value of all price indices to a certain crude oil price shock; see Figure A1.

We implement the impulse response function between each crude oil and eight price indices following the steps of Section 3.2.1. To explore the evolution of the direct impact of crude oil price shocks on each price index, we extract the first 15 months and their corresponding response values, shown in Figure 3 and Appendix Table A1. It can be observed that the evolution trends of the response value of each price index to the four kinds of oil price shocks are similar. However, the degree and duration of the impacts are different from price index to price index. Crude oil price acts as one of the influencing factors on the Chinese price level, and the transmission mechanism of crude oil price fluctuation to Chinese commodities price is cost driven. Due to the long and complex industrial chain of the oil industry, the influence of oil price shocks on different links of industrial chains is different, causing the price indices to be affected differently. The detailed analysis is as follows.

**Figure A1:**
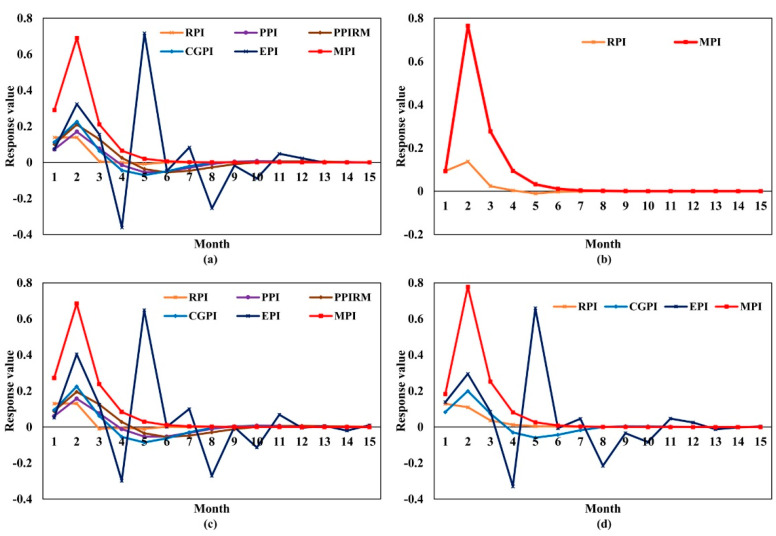
The response of price indices to the shocks of four kinds of crude oil prices. The *X*-axis represents the time from the first month to the fifteenth month, and the *Y*-axis is the response value. (**a**) Response value to Brent crude oil shocks; (**b**) response value to WTI crude oil shocks; (**c**) response value to Dubai crude oil shocks; (**d**) response value to Daqing crude oil shocks.

First, the impact of crude oil price shocks on MPI and EPI is mainly due to the increasing transportation cost of international trade and the increasing cost of commodities production. As expected, there is a sustained and positive reaction in MPI (Figure 3a). Compared to other price indices, the response values of MPI to the shock in four crude oil prices are the largest in the first three months (see Appendix Figure A1). Furthermore, the responses to Brent, WTI, Dubai, and Daqing in the second month reach a peak of 0.6901, 0.7649, 0.6846, and 0.7778, respectively. The impact effects begin to subside right after the second month and die out in the eighth month. EPI (Figure 3d) cannot be directly affected by WTI oil price, and the responses of EPI to Brent, Dubai, and Daqing oil price fluctuations are more volatile. Specifically, EPI shows a positive response in the first two months. Then, it tends to decrease quickly, and the responses turn negative in the fourth month. However, the direct impacts of oil price shocks on EPI turn positive again and reach a peak of 0.6400 in the fifth month. The direct impacts could last for fifteen months.

Second, crude oil can be industrially fractionated into various chemical materials and oil and gas products, which are the raw materials of industrial products. Thus, the crude oil price fluctuations could be transmitted to CGPI, PPI, and PPIRM directly. From Figure 3c,e,f, we can conclude that the reaction of CGPI, PPI, and RPIRM to the shock in oil price is similar. Furthermore, there is an immediate and positive response of the three price indices to a onetime shock in oil price. The impacts of oil price fluctuations are largest in the second month, after which the impacts decline sustainedly and turn negative. In the fifth or sixth month, the negative impacts reach the maximum. Furthermore, the degree of impact of Brent and Dubai oil price on CGPI is larger than that of Daqing, and PPI and PPIRM could only be directly affected by Brent and Dubai oil prices. The duration of the direct impacts on CGPI, PPI, and PPIRM is about 11, 12, and 9 months, respectively.

Third, crude oil can also be industrially fractionated into refined oil products that can be directly consumed by consumers. When the price transmission mechanism is unobstructed, the influence of crude oil price fluctuation could transmit from upstream to downstream; that is, the consumer market could be directly affected by crude oil price fluctuations. However, based on the Granger causality test, the causal relationships from crude oil to CPI are not statistically significant, proving that the fluctuation transmission of crude oil prices is not smooth. This might be because the Chinese government formulates a pricing mechanism for refined oil products and takes a series of measures to stabilize the consumer market, decreasing the impact of crude oil price shocks on CPI. From Figure 3b, it is observed that the shocks of four crude oil prices could be transmitted to RPI. The responses of RPI are larger in the first two months, after which the responses decline sharply and fade away over time. The direct effects are significant until the sixth month.

We also sum the absolute values of the direct impacts of crude oil price shocks on each price index in the first 15 months, as shown in Table 1. It can be figured out that the direct impacts on RPI are the lowest, while the direct impacts on EPI are the largest. The impact of crude oil price fluctuations on trade costs could be significantly reflected in the import and export price index. Furthermore, Chinese price indices are more sensitive to Brent and Daqing crude oil price shocks.

### 4.2. The Indirect Effects of Crude Oil Price Shocks

**Figure 4:**
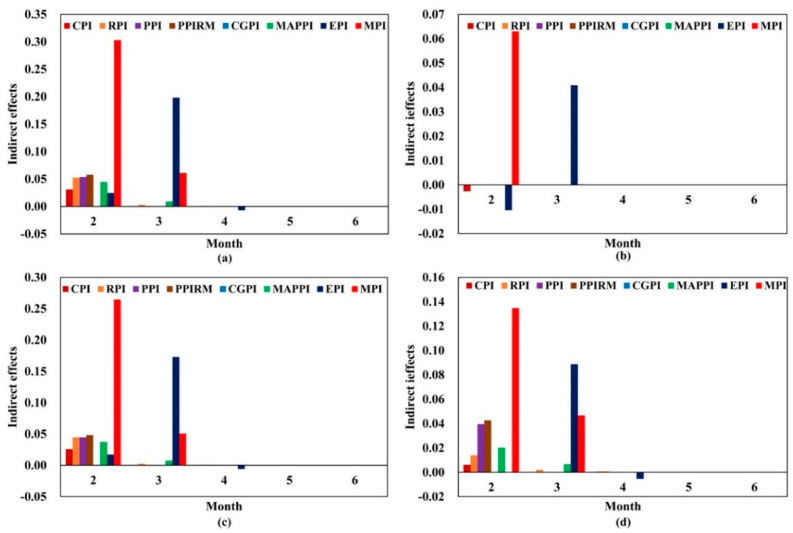
The indirect effects of the four kinds of crude oil shocks on price indices. The *X*-axis represents the time from the second month to the seventh month, and the *Y*-axis is the indirect impact value. (**a**) Indirect effect value of Brent crude oil shocks; (**b**) indirect effect value of WTI crude oil shocks; (**c**) indirect effect value of Dubai crude oil shocks; (**d**) indirect effect value of Daqing crude oil shocks.

The result of Section 3.1 is the response value of each price index to crude oil shocks. After a positive shock in crude oil price, not all of the price indices are directly influenced. Since there are transmission relationships among price indices, the crude oil price shocks could further impact more price indices. For example, crude oil price fluctuation can transmit to PPI by influencing the price of industrial raw materials, and due to the supply–demand relations, the fluctuation of the PPI could further impact consumer prices, such as CPI. In this paper, we define that there is an indirect impact from crude oil to CPI. Moreover, since the direct impacts vary over time, the indirect effects also change over time. Thus, we calculate the indirect impact of crude oil price shocks on price indices based on Section 3.2.2. The indirect impacts of the shocks of four kinds of crude oil prices on price indices from the second month to the fifteenth month are shown in Appendix Table A2. To offer more detailed and finer observations on the indirect impacts over time, we abstract the indirect effects from the second month to the sixth month and draw Figure 4. It can be concluded that the evolution trends of the indirect effects are different from those of the direct effects.

For MPI, the indirect impacts of oil price shocks are positive (Figure 4). The indirect impacts of Brent, WTI, Dubai, and Daqing reach a peak of 0.3031, 0.0630, 0.2646, and 0.1348, respectively, in the second month, meaning a positive shock of one standard deviation in the four crude oil prices has an indirect positive impact on MPI in the second month, with values of 0.5469, 0.3875, 0.4772, and 0.4654, respectively. The indirect impact could last for only two months. For EPI, in the second month, the impacts of Brent and Dubai are positive, while the impacts of WTI and Daqing are negative. The indirect impacts of four kinds of crude oil price shocks are positive in the third month with a peak of 0.1981, 0.0409, 0.1729, and 0.0887, respectively. However, the indirect impacts turn negative in the fourth month, after which the impacts die out.

For CGPI, only Daqing crude oil price fluctuations could indirectly affect it, and the impacts are too small that they could be ignored. For PPI and PPIRM, Brent and Dubai crude oil price fluctuations could only affect them in the second month with the impact degree exceeding 0.044. Although Daqing could influence them in the long run, the impacts are small. Thus, the duration of the indirect impact of crude oil price shocks on PPI and PPIRM is only one month. Furthermore, the indirect impacts on RPI are the largest in the second month, and the impacts could last until the fourth month.

Through analyzing the indirect effects, we find that some price indices that are not directly affected by crude oil price shocks are indirectly impacted, such as CPI and MAPPI. For MAPPI, Brent and Dubai crude oil price fluctuations could only indirectly affect it in the second and third months. Although a positive shock to Daqing oil price could indirectly affect MAPPI for a long period, the impacts are small after the third month and can be ignored. For CPI, the impacts of the shocks of four oil prices are significant only in the second month, with a value of less than 0.0310.

We also sum the absolute values of the indirect impacts of crude oil price shocks on each price index from the second month to the fifteenth month, as shown in Table 2. Compared to other price indices, the indirect impacts on MPI are the largest, followed by EPI and PPIRM. Furthermore, crude oil price shocks have no direct impact on MAPPI, while the indirect impacts are much higher. On the contrary, the indirect impacts on CPI are the lowest. This might be because the price shocks of crude oil are gradually transmitted along the industrial chain, and the price transmission is gradually weakened from upstream to downstream with the reduction of industry concentration. CPI is at the downstream of the crude oil industry chain and is weakly affected by crude oil price fluctuations.

### 4.3. The Total Impacts of Crude Oil Price Shocks

**Figure 5:**
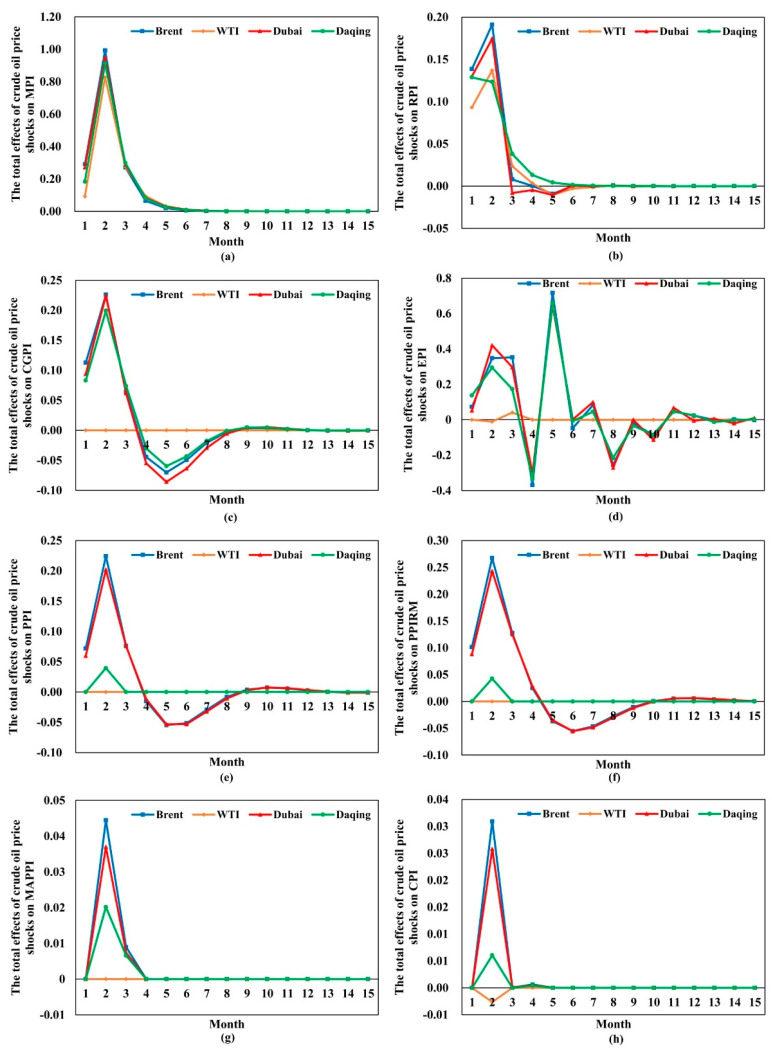
The total impact of crude oil price shocks on price indices. (**a**–**h**) represent the total effects of crude oil price shocks on MPI, RPI, CGPI, EPI, PPI, PPIRM, MAPPI, and CPI, respectively.

To deepen the understanding of the total impacts of a positive shock to oil price on each price index over time, we calculate the sum of each month’s direct and indirect impacts, as shown in Figure 5, which reveals the impact degree and duration. The duration of the total impacts is similar to the direct impacts, and the impacts on EPI last the longest with about 15 months. Furthermore, the impact direction of crude oil price fluctuation on EPI is highly uncertain. Except for EPI, most of the total impacts reach their peak in the second month.

Based on Table 1 and Table 2, we sum the absolute values of the direct and indirect impacts of crude oil price shocks on each price index and calculate the proportion of the indirect impacts for the total impacts, as shown in Table 3. The total impacts on EPI and MPI are the largest, and impacts on CPI are the lowest. Taking a further analysis, the proportions range from 0.03% to 100.00%. Except for 100%, the proportions of the indirect impacts on MPI are larger than other price indices. Furthermore, it could be concluded that the proportions of the indirect impact of Brent oil price shocks are higher than other oil price shocks. Our results prove that the indirect influence cannot be ignored when analyzing the influence of crude oil price fluctuation on the Chinese price level.

## 5. Conclusions

In this paper, we creatively combine the complex network method and econometric methods to reveal the direct and indirect impacts of crude oil price shocks on Chinese price indices over time. The degree and duration of the impacts of crude oil price shocks on each price index are analyzed. Some conclusions and policy implications could be summarized as follows.

(1) In the sample period, not all the Chinese price indices are directly affected by crude oil price shocks, such as CPI and MAPPI, while they can be indirectly influenced, although the impacts are relatively weak. This indicates that the transmission mechanism of oil price fluctuations to the Chinese price level is obstructed, and a series of price stabilizing measures taken by China to resist cost-driven inflation is effective. Thus, consumers and farmers do not worry too much about the impacts of oil price volatility on their production and consumption activities. Furthermore, the direct and total response of EPI to crude oil price shock is higher and more volatile with uncertain response direction. The influence of crude oil price shock on MPI is significantly positive and can last for about 8 months. Companies whose main business is mainly for exporting and importing products (exporters and importers) should pay more focus to the fluctuations of crude oil price and adjust production, import and export plans in time, reducing the impacts of oil price shocks. Furthermore, the responses of CGPI, PPI, and PPIRM are similar. Thus, policymakers could take similar measures for monitoring CGPI, PPI, and PPPRM when crude oil price fluctuates. Researchers have found that energy intensity is an essential factor impacting the inter-industry differences in oil shocks [35]; thus, energy-intensive companies and producers should pay more attention to the impacts of oil price shocks.
(2) The proportions of the indirect impacts of crude oil price shocks on price indices for the total impacts are much higher, ranging from 0.03% to 100% with an average of 44.21%. Furthermore, the indirect impact could only last for about two or three months. Therefore, when measuring the impact of crude oil price fluctuation on the price indices, market regulators need to consider its indirect impacts. Otherwise, the impacts of crude oil price fluctuations could be underestimated, resulting in errors in the formulation of price stabilization strategies by the government; thus, we could not effectively curb the impact of crude oil price fluctuations on the Chinese price level.
(3) Oil price shocks can affect Chinese price levels in the short run, and the impacts fade out in the long run. Furthermore, the duration of the total impacts is similar to the direct impacts and varies from price index to price index. Specifically, the total impacts of the crude oil price shock on EPI last the longest, about 15 months, while the impacts on CPI and MAPPI are not significant after the third month. Furthermore, except for EPI, the responses of all price indices reach the maximum in the second month after the oil price shocks. Thus, to not miss the best time to monitor the price level, policymakers need to recognize the evolution trend of the impacts of crude oil price shocks and formulate measures to intervene in the transmission path from crude oil to Chinese commodities promptly.
(4) The shocks of different crude oil prices have different impacts on Chinese price levels. Chinese price indices are more sensitive to Brent oil price fluctuations, and the effects of WTI oil price fluctuation are much lower. Chinese policymakers should focus more on the fluctuation of Brent oil prices.
  This paper offers a method to research the direct and indirect influences of crude oil price shocks on the price level. It can compensate for the data lag of the input–output table and the multiple-collinearity of econometric methods. However, there are many factors influencing the Chinese price level, such as exchange rate, GDP, etc. In the future, we will consider more influencing factors to analyze the direct and indirect impacts on Chinese commodities. Furthermore, we will analyze the dynamic transmission effects from crude oil to the price indices over time based on a sliding window approach. It is also an interesting topic to investigate the contagion effects [41] and identify the dynamic transmission path from crude oil to the price indices.

## Figures and Tables

**Table 1:** The total direct impacts on each price index.

	Brent	WTI	Dubai	Daqing
MPI	1.2850	1.2756	1.3224	1.3324
EPI	2.1980	—	2.1220	1.9811
CGPI	0.6007	—	0.6339	0.5207
PPI	0.4994	—	0.4767	—
PPIRM	0.6588	—	0.6372	—
RPI	0.2940	0.2729	0.2885	0.2931
CPI	—	—	—	—
MAPPI	—	—	—	—

Note: “—” means that the corresponding price index could not be directly impacted by crude oil price shocks.

**Table 2:** The total indirect impacts on price indices.

	Brent	WTI	Dubai	Daqing
MPI	0.3642	0.0633	0.3155	0.1816
EPI	0.2297	0.0513	0.1960	0.0942
CGPI	—	—	—	0.0002
PPI	0.0536	—	0.0446	0.0396
PPIRM	0.0579	—	0.0481	0.0427
RPI	0.0563	0.0001	0.0476	0.0166
CPI	0.0316	0.0026	0.0264	0.0065
MAPPI	0.0534	—	0.0444	0.0268

Note: “—” means that the corresponding price index could not be indirectly impacted by crude oil price shocks.

**Table 3:** The proportion of the indirect impact on total impacts.

	Total Impacts				Proportion			
	Brent	WTI	Dubai	Daqing	Brent	WTI	Dubai	Daqing
MPI	1.6492	1.3389	1.6379	1.5140	22.08%	4.72%	19.26%	11.99%
EPI	2.4277	0.0513	2.3180	2.0753	9.46%	100.00%	8.46%	4.54%
CGPI	0.6007	—	0.6339	0.5209	—	—	—	0.04%
PPI	0.5530	—	0.5213	0.0396	9.70%	—	8.56%	100.00%
PPIRM	0.7167	—	0.6853	0.0427	8.07%	—	7.03%	100.00%
RPI	0.3503	0.2730	0.3362	0.3098	16.07%	0.03%	14.17%	5.37%
CPI	0.0316	0.0026	0.0264	0.0065	100.00%	100.00%	100.00%	100.00%
MAPPI	0.0534	—	0.0444	0.0268	100.00%	—	100.00%	100.00%

Note: “—” means that the corresponding price index could not be impacted by crude oil price shocks.

## Data Availability

The crude oil spot price of WTI, Brent, Dubai, and Daqing (China) are obtained from the Wind Database (http://www.wind.com (accessed on 1 March 2022)) and the Energy Information Admin-istration (http://www.eia.gov (accessed on 1 March 2022)). The price indices we use in this paper are downloaded from the Wind Database (accessed on 1 March 2022).

## Appendix A

**Table A1:** The proportion of the indirect impact of total impacts.

	CPI				RPI				PPI				PPIRM			
Time	Brent	WTI	Dubai	Daqing	Brent	WTI	Dubai	Daqing	Brent	WTI	Dubai	Daqing	Brent	WTI	Dubai	Daqing
1	-	-	-	-	0.1385	0.0931	0.1299	0.1288	0.0720	-	0.0599	-	0.1015	-	0.0883	-
2	-	-	-	-	0.1387	0.1369	0.1301	0.1095	0.1706	-	0.1574	-	0.2097	-	0.1951	-
3	-	-	-	-	0.0052	0.0237	−0.0104	0.0361	0.0763	-	0.0757	-	0.1278	-	0.1254	-
4	-	-	-	-	−0.0008	0.0033	−0.0056	0.0124	−0.0151	-	−0.0117	-	0.0249	-	0.0278	-
5	-	-	-	-	−0.0091	−0.0106	−0.0105	0.0042	−0.0548	-	−0.0533	-	−0.0371	-	−0.0349	-
6	-	-	-	-	0.0003	−0.0029	0.0007	0.0015	−0.0513	-	−0.0529	-	−0.0553	-	−0.0556	-
7	-	-	-	-	−0.0004	−0.0012	−0.0004	0.0005	−0.0294	-	−0.0324	-	−0.0463	-	−0.0484	-
8	-	-	-	-	0.0007	0.0007	0.0007	0.0002	−0.0083	-	−0.0107	-	−0.0276	-	−0.0300	-
9	-	-	-	-	−0.0001	0.0003	−0.0002	0.0001	0.0039	-	0.0029	-	−0.0104	-	−0.0121	-
10	-	-	-	-	0.0001	0.0002	0.0000	0.0000	0.0073	-	0.0076	-	0.0006	-	−0.0000	-
11	-	-	-	-	−0.0001	−0.0000	−0.0001	0.0000	0.0057	-	0.0065	-	0.0054	-	0.0055	-
12	-	-	-	-	0.0000	−0.0000	0.0000	0.0000	0.0026	-	0.0033	-	0.0057	-	0.0063	-
13	-	-	-	-	−0.0000	−0.0000	−0.0000	0.0000	0.0002	-	0.0006	-	0.0041	-	0.0047	-
14	-	-	-	-	0.0000	0.0000	0.0000	0.0000	−0.0009	-	−0.0009	-	0.0020	-	0.0024	-
15	-	-	-	-	−0.0000	0.0000	−0.0000	0.0000	−0.0010	-	−0.0011	-	0.0004	-	0.0006	-
	CGPI				MAPPI				EPI				MPI			
Time	Brent	WTI	Dubai	Daqing	Brent	WTI	Dubai	Daqing	Brent	WTI	Dubai	Daqing	Brent	WTI	Dubai	Daqing
1	0.1127	-	0.0938	0.0831	-	-	-	-	0.0727	-	0.0517	0.1379	0.2904	0.0922	0.2713	0.1836
2	0.2261	-	0.2245	0.1993	-	-	-	-	0.3235	-	0.4036	0.2945	0.6901	0.7649	0.6846	0.7778
3	0.0638	-	0.0616	0.0738	-	-	-	-	0.1548	-	0.1244	0.0846	0.2106	0.2764	0.2378	0.2518
4	−0.0445	-	−0.0549	−0.0301	-	-	-	-	−0.3616	-	−0.2986	−0.3294	0.0649	0.0940	0.0835	0.0809
5	−0.0702	-	−0.0860	−0.0597	-	-	-	-	0.7170	-	0.6482	0.6591	0.0200	0.0318	0.0293	0.0260
6	−0.0494	-	−0.0636	−0.0435	-	-	-	-	−0.0473	-	0.0011	−0.0069	0.0062	0.0108	0.0103	0.0084
7	−0.0209	-	−0.0294	−0.0179	-	-	-	-	0.0834	-	0.0998	0.0453	0.0019	0.0036	0.0036	0.0027
8	−0.0027	-	−0.0055	−0.0010	-	-	-	-	−0.2546	-	−0.2707	−0.2154	0.0006	0.0012	0.0013	0.0009
9	0.0038	-	0.0044	0.0048	-	-	-	-	−0.0180	-	−0.0006	−0.0343	0.0002	0.0004	0.0004	0.0003
10	0.0036	-	0.0052	0.0042	-	-	-	-	−0.0890	-	−0.1136	−0.0836	0.0001	0.0001	0.0002	0.0001
11	0.0016	-	0.0027	0.0018	-	-	-	-	0.0476	-	0.0682	0.0462	0.0000	0.0000	0.0001	0.0000
12	0.0001	-	0.0005	0.0001	-	-	-	-	0.0230	-	−0.0051	0.0249	0.0000	0.0000	0.0000	0.0000
13	−0.0005	-	−0.0006	−0.0005	-	-	-	-	−0.0012	-	0.0063	−0.0124	0.0000	0.0000	0.0000	0.0000
14	−0.0004	-	−0.0007	−0.0005	-	-	-	-	0.0024	-	−0.0202	−0.0033	0.0000	0.0000	0.0000	0.0000
15	−0.0002	-	−0.0004	−0.0002	-	-	-	-	−0.0018	-	0.0098	0.0034	0.0000	0.0000	0.0000	0.0000

Note: “-” means that the corresponding price index could not be directly impacted by crude oil price shocks.

**Table A2:** The indirect impacts of four kinds of crude oil price shocks on price indices from the second to fifteenth month.

	CPI				RPI				PPI				PPIRM			
Time	Brent	WTI	Dubai	Daqing	Brent	WTI	Dubai	Daqing	Brent	WTI	Dubai	Daqing	Brent	WTI	Dubai	Daqing
2	0.0309	−0.0026	0.0258	0.0060	0.0525	-	0.0445	0.0139	0.0536	-	0.0446	0.0395	0.0579	-	0.0481	0.0426
3	0.0000	-	0.0001	−0.0000	0.0028	−0.0001	0.0023	0.0020	-	-	-	-	-	-	-	-
4	0.0006	0.0000	0.0005	0.0004	0.0011	-	0.0009	0.0008	-	-	-	0.0000	-	-	-	0.0001
5	0.0000	-	0.0000	0.0000	0.0000	0.0000	0.0000	−0.0000	-	-	-	−0.0000	-	-	-	−0.0000
6	−0.0000	−0.0000	−0.0000	0.0000	−0.0000	-	−0.0000	0.0000	-	-	-	−0.0000	-	-	-	0.0000
7	−0.0000	-	−0.0000	−0.0000	−0.0000	−0.0000	−0.0000	−0.0000	-	-	-	−0.0000	-	-	-	0.0000
8	0.0000	0.0000	0.0000	−0.0000	0.0000	-	0.0000	0.0000	-	-	-	0.0000	-	-	-	−0.0000
9	0.0000	-	0.0000	−0.0000	0.0000	0.0000	0.0000	−0.0000	-	-	-	−0.0000	-	-	-	0.0000
10	−0.0000	−0.0000	−0.0000	0.0000	−0.0000	-	−0.0000	0.0000	-	-	-	−0.0000	-	-	-	−0.0000
11	−0.0000	-	−0.0000	−0.0000	−0.0000	−0.0000	−0.0000	−0.0000	-	-	-	0.0000	-	-	-	−0.0000
12	0.0000	0.0000	0.0000	−0.0000	0.0000	-	0.0000	0.0000	-	-	-	0.0000	-	-	-	−0.0000
13	0.0000	-	0.0000	−0.0000	0.0000	0.0000	0.0000	0.0000	-	-	-	−0.0000	-	-	-	0.0000
14	0.0000	0.0000	0.0000	0.0000	−0.0000	-	−0.0000	0.0000	-	-	-	−0.0000	-	-	-	0.0000
15	−0.0000	-	−0.0000	0.0000	0.0000	0.0000	0.0000	0.0000	-	-	-	−0.0000	-	-	-	0.0000
	CGPI				MAPPI				EPI				MPI			
Time	Brent	WTI	Dubai	Daqing	Brent	WTI	Dubai	Daqing	Brent	WTI	Dubai	Daqing	Brent	WTI	Dubai	Daqing
2	-	-	-	-	0.0444	-	0.0370	0.0202	0.0246	−0.0104	0.0173	−0.0001	0.3031	0.0630	0.2646	0.1348
3	-	-	-	0.0002	0.0089	-	0.0074	0.0066	0.1981	0.0409	0.1729	0.0887	0.0611	0.0002	0.0508	0.0467
4	-	-	-	−0.0000	-	-	-	0.0000	−0.0070	−0.0000	−0.0058	−0.0054	0.0000	−0.0000	0.0000	0.0001
5	-	-	-	0.0000	-	-	-	−0.0000	−0.0000	0.0000	−0.0000	0.0000	−0.0000	−0.0000	−0.0000	0.0000
6	-	-	-	0.0000	-	-	-	0.0000	0.0000	0.0000	0.0000	−0.0000	0.0000	0.0000	0.0000	−0.0000
7	-	-	-	−0.0000	-	-	-	−0.0000	0.0000	0.0000	0.0000	−0.0000	0.0000	0.0000	0.0000	−0.0000
8	-	-	-	0.0000	-	-	-	0.0000	0.0000	0.0000	0.0000	0.0000	−0.0000	−0.0000	−0.0000	−0.0000
9	-	-	-	0.0000	-	-	-	−0.0000	−0.0000	−0.0000	−0.0000	−0.0000	−0.0000	−0.0000	−0.0000	0.0000
10	-	-	-	0.0000	-	-	-	−0.0000	0.0000	0.0000	0.0000	−0.0000	0.0000	0.0000	0.0000	−0.0000
11	-	-	-	0.0000	-	-	-	−0.0000	0.0000	0.0000	0.0000	−0.0000	0.0000	0.0000	0.0000	0.0000
12	-	-	-	−0.0000	-	-	-	−0.0000	0.0000	0.0000	0.0000	0.0000	−0.0000	−0.0000	−0.0000	0.0000
13	-	-	-	−0.0000	-	-	-	−0.0000	−0.0000	−0.0000	−0.0000	0.0000	−0.0000	−0.0000	−0.0000	−0.0000
14	-	-	-	−0.0000	-	-	-	0.0000	0.0000	0.0000	0.0000	0.0000	0.0000	0.0000	0.0000	0.0000
15	-	-	-	−0.0000	-	-	-	0.0000	0.0000	0.0000	0.0000	−0.0000	−0.0000	−0.0000	−0.0000	0.0000

Note: “-” means that the corresponding price index could not be indirectly impacted by crude oil price shocks in a certain month.
